# Permeability of the peroxisomal membrane: lessons from the glyoxylate cycle

**DOI:** 10.3389/fphys.2013.00204

**Published:** 2013-08-14

**Authors:** Markus Kunze, Andreas Hartig

**Affiliations:** ^1^Department of Pathobiology of the Nervous System, Center for Brain Research, Medical University of ViennaVienna, Austria; ^2^Department of Biochemistry and Cell Biology, Max F. Perutz Laboratories, University of ViennaVienna, Austria

**Keywords:** glyoxylate, glyoxylate cycle, peroxisomes, pore forming protein, membrane permeability, metabolite transfer, metabolon, photorespiration

## Abstract

Glyoxylate serves as intermediate in various metabolic pathways, although high concentrations of this metabolite are toxic to the cell. In many organisms glyoxylate is fed into the glyoxylate cycle. Enzymes participating in this metabolism are located on both sides of the peroxisomal membrane. The permeability of this membrane for small metabolites paves the way for exchange of intermediates between proteins catalyzing consecutive reactions. A model, in which soluble enzymes accumulate in close proximity to both ends of pore-like structures forming a transmembrane metabolon could explain the rapid and targeted exchange of intermediates. The metabolites passing the membrane differ between the three model organisms *Saccharomyces cerevisiae*, *Arabidopsis thaliana*, and *Candida albicans*, which reflects the ease of evolutionary adaptation processes whenever specific transporter proteins are not involved. The atypical permeability properties of the peroxisomal membrane together with a flexible structural arrangement ensuring the swift and selective transport across the membrane might represent the molecular basis for the functional versatility of peroxisomes.

## Introduction

Peroxisomes are defined as organelles encasing the metabolism of H_2_O_2_. This highly reactive molecule is generated by several oxidative reactions and degraded inside the organelle by abundant amounts of catalase. In addition, peroxisomes fulfill a number of important metabolic functions for eukaryotic cells requiring an active communication between the peroxisomal lumen and the cytosol or other organelles. Major functions include the β-oxidation of fatty acids, parts of the glyoxylate cycle and parts of the photorespiration (Hu et al., 2012; Waterham and Wanders, 2012). For β-oxidation fatty acids have to enter peroxisomes, and the resulting acetyl-CoA is distributed throughout the cells. Photorespiration in plants involves mitochondria, chloroplasts and peroxisomes and therefore requires extensive metabolite exchange. A key intermediate shared by the photorespiration and the glyoxylate cycle is glyoxylate, usually generated and metabolized inside peroxisomes. The glyoxylate cycle was originally considered a metabolic process localized to peroxisomes (Breidenbach and Beevers, 1967). This localization was rationalized as a means to increase the efficiency of the flux of intermediates. However, the finding that parts of the whole cycle are extra-peroxisomal in the yeast *Saccharomcyes cerevisiae* and in the plant *Arabidopsis thaliana* indicated, that a functional glyoxylate cycle requires the transfer of various metabolites across the peroxisomal membrane, too (Minard and McAlister-Henn, 1991; Courtois-Verniquet and Douce, 1993; Taylor et al., 1996; Kunze et al., 2002; Pracharoenwattana et al., 2007). The nature of the molecules crossing the peroxisomal membrane varies with the organism under study.

The single membrane separating the peroxisomal matrix from the surrounding was shown to be permeable only for small molecules such as tri- and dicarboxylates and amino acids (Antonenkov et al., 2009). Specific transporters have not yet been identified but features compatible with pore-like structures were demonstrated (Verleur and Wanders, 1993; Antonenkov and Hiltunen, 2006).

In this review we will address the questions how and where glyoxylate is generated and how metabolites of the two most prominent pathways partially localized to peroxisomes, the glyoxylate cycle and the photorespiration, are thought to cross the membrane on their way in and out of the peroxisomes.

## Peroxisomes and glyoxylate metabolism

**Figure 1:**
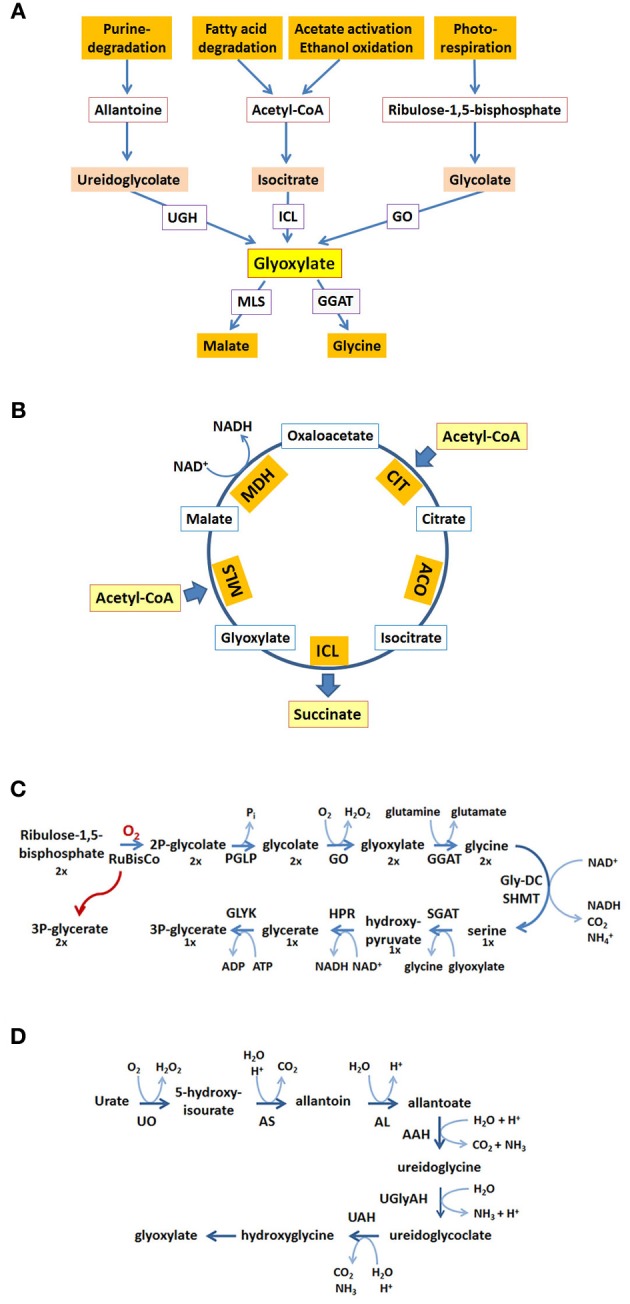
**Glyoxylate generation and consumption. (A)** Glyoxylate is generated from different precursor molecules and converted into stable metabolites for further utilization. The proteins involved are ureidoglycolate hydrolase (UGH), isocitrate lyase (ICL), glycolate oxidase (GO), malate synthase (MLS), glutamine:glyoxylate aminotransferase (GGAT). **(B)** In the glyoxylate cycle two acetyl-CoA are condensed to succinate and 2 CoA (not shown). The proteins involved are citrate synthase (CIT), aconitase (ACO), isocitrate lyase (ICL), malate synthase (MLS), malate dehydrogenase (MDH). **(C)** The generation and consumption of glyoxylate in the photorespiratory process. The stoichiometry of the reaction sequence to obtain three molecules of 3-phospho-glycerate from two oxygenation products of ribulose-1,5-bisphosphate is indicated by the numbers below the molecules (1×, 2×). The proteins involved are ribulose-bisphosphate-carboxylase-oxygenase (RuBisCO), phosphoglycolate phosphatase (PGLP), glycolate oxidase (GO), glutamine:glyoxylate aminotransferase (GGAT), glycine-decarboxylase (GlyDC), serine-hydroxymethyltransferase (SHMT), serine:glyoxylate aminotransferase (SGAT), hydroxypyruvate reductase (HPR), glycerate kinase (GLYK). **(D)** The reactions in the purine degradation pathway leading to glyoxylate. The proteins involved are urate oxidase (UO), allantoin synthase (HIU-hydrolase + OHCU decarboxylase) (AS), allantoinase (alantoin amidohydrolase) (AL), allantoate amidohydrolase (AAH), ureidoglycine aminohydrolase (UGlyAH), ureidoglycolate amidohydrolase (UAH).

Inside peroxisomes the accumulation of glyoxylate is prevented by conversion into glycine making use of a transaminase reaction or by condensation with acetyl-CoA into malate catalyzed by malate synthase (MLS), one of the key enzymes of the glyoxylate pathway (for reviews see Wanders and Waterham, 2006; Theodoulou and Eastmond, 2012). Glyoxylate can be produced from different precursor molecules (Figure 1A). Cleavage of isocitrate generates glyoxylate and succinate in the glyoxylate cycle (Figure 1B). The oxidation of glycolate to glyoxylate is catalyzed by glycolate oxidase being part of the photorespiratory process generating H_2_O_2_ within peroxisomes (Figure 1C). Another possible source of glyoxylate is the degradation of purines in those organisms metabolizing the intermediate uric acid (Figure 1D). Moreover, in mammals glyoxylate may represent a degradation product of hydroxyproline originally derived from collagen (Salido et al., 2012) or alternatively, may be the result of a conversion from glycine catalyzed by D-amino acid oxidase generating H_2_O_2_ (Ohide et al., 2011).

The glyoxylate cycle allows the formation of 4-carbon units from 2-carbon units (Figure 1B). The resulting succinate serves to replenish the tricarboxylic acid (TCA) cycle representing the major collector and distributor of small carbon units. Alternatively, succinate or its follow-up product oxaloacetate serves as precursor for many biosynthetic processes. This biosynthetic pathway is absent in all animals except nematodes (Kondrashov et al., 2006). Acetyl-CoA fed into the glyoxylate cycle can be derived from different sources, such as β-oxidation of fatty acids, degradation of amino acids or in case of microbial organisms from external carbon sources such as ethanol or acetate. The glyoxylate cycle shares a series of three enzymatic activities with the TCA-cycle, namely malate dehydrogenase (MDH), citrate synthase (CIT), and aconitase (ACO) activity. The two unique activities, isocitrate lyase (ICL) and malate synthase (MLS) generate and consume the name-giving metabolite glyoxylate. The cleavage of isocitrate bypasses the decarboxylation reactions and the synthase reaction leads to the net-condensation of acetyl-CoA units. In many organisms these activities can be carried out by two or more isoenzymes with different localization signals and different expression patterns catalyzing the respective reactions.

Photorespiration is required in all photosynthetic organisms and serves as carbon recovery system (Maurino and Peterhansel, 2010). Oxygenation of ribulose-1,5-bisphosphate by ribulose-bisphosphate-carboxylase-oxygenase (RuBisCO) competes with CO_2_ fixation upon high partial oxygen pressure leading to the formation of 2-phosphoglycolate and 3-phosphoglycerate in the chloroplast (Figure 1C). 3-phosphoglycerate is channeled into the Calvin cycle and 2-phosphoglycolate is recycled into 3-phosphoglycerate in a series of reactions involving peroxisomes and mitochondria. In peroxisomes glycolate is oxidized to glyoxylate generating H_2_O_2_, which is immediately decomposed by catalase. Glyoxylate is transaminated to glycine using either serine or glutamine as nitrogen source and further transported to the mitochondria. Here, two glycines are converted into one serine which is now returned into peroxisomes. The peroxisomal serine:glyoxylate aminotransferase and hydroxypyruvate reductase convert serine into glycerate which is transferred to chloroplasts, where it enters the Calvin cycle in the form of 3-phosphoglycerate. In summary, two glycolate molecules are consumed to produce glycerate and CO_2_ and the generation of two H_2_O_2_ is confined to peroxisomes.

The degradation pathway of purines leads to uric acid, which in many organisms is further metabolized within peroxisomes to allantoine and allantoic acid (Figure 1D). In some animals the allantoicase activity leads to the cleavage of allantoic acid into urea and glyoxylate (Hayashi et al., 2000). In plants, some fungi and bacteria allantoic acid is further processed within the endoplasmic reticulum into ureidoglycine and ureidoglycolate, both giving rise to glyoxylate upon degradation (Werner and Witte, 2011). The glyoxylate derived from purine degradation is supposedly converted into glycine or condensed with acetyl-CoA catalyzed by a MLS activity providing the versatile metabolite malate. In essence, the heterocyclic core structure is converted into a nitrogen containing product and glyoxylate. The latter is recycled into biosynthetic processes in all organisms, the nitrogen containing product is either excreted (animals) or recycled (plants).

## Enzymes allocated to the glyoxylate cycle

Due to the fact that three of the five enzymatic activities contributing to the glyoxylate cycle are shared with the TCA cycle and additional enzymatic activities have been described an assignment of individual proteins to the glyoxylate cycle is not trivial. Some of the encoded proteins can be excluded due to an inappropriate expression pattern, but for some of the proteins only the phenotype of cells lacking the respective activity is a convincing argument for the function in the glyoxylate cycle. As the situation in the yeast *S. cerevisiae* has been analysed in detail previously (Kunze et al., 2006), we will only summarize the most important information and then concentrate on more recent results obtained in the thale cress *A. thaliana* as the plant model organism and in the human opportunistic fungus *Candida albicans*. The corresponding proteins are listed in Table 1.

**Table 1:** **Enzymatic activities required for the glyoxylate cycle**.

**Enzyme**	***Saccharomyces cerevisiae***			***Candida albicans***			***Arabidopsis thaliana***		
	**Protein**	**Gene**	**Targeting information**	**Protein**	**Gene**	**Targeting information**	**Protein**	**Gene**	**Targeting information**
Isocitrate lyase	**ICL1**	YER065C	Cytosolic	ICL1	CaO19.14134	Peroxisomal	ICL	At3g21720	Peroxisomal
	ICL2	YPR006C	Mitochondrial						
Malate synthase	**MLS1**	YNL117W	Peroxisomal	MLS1	CaO19.12296	Peroxisomal	MLS	At5g03860	Peroxisomal
	DAL7	YIR031C	PTS1						
Malate	MDH1	YKL085W	Mitochondrial	MDH1	CaO19.12072	MITO	pMDH1	At2g22780	Peroxisomal
dehydrogenase	**MDH2**	YOL126C	Cytosolic	MDH2	CaO19.7481		pMDH2/MDHG1	At5g09660	Peroxisomal
	MDH3	YDL078C	Peroxisomal	MDH3	CaO19.1278	PTS1	mDH3/MDHM1	At1g53240	Mitochondrial
							mDH4/MDHM2	At3g15020	Mitochondrial
							MDH5/MDHC1	At1g04410	
							MDH6/MDHC2	At5g43330	
							MDH7/MDHC3	AT5G56720	
							cMDH8/MDHP1	At3g47520	Chloroplast
Citrate synthase	CIT1	YNR001C	Mitochondrial	CIT1	CaO19.11871	Mitochondrial	CSY1	At3g58740	PTS2
	**CIT2**	YCR005C	Peroxisomal		Q59ZZ5		CSY2	At3g58750	Peroxisomal
	CIT3	YPR001W	Mitochondrial				CSY3	At2g42790	Peroxisomal
							CSY4	At2g44350	Mitochondrial
							CSY5	At3g60100	Mitochondrial
Aconitase	**ACO1**	YLR304C	Mitochondrial	ACO1	CaO19.13742	MITO	ACO1	At4g35830	Mitochondrial
	ACO2	YJL200C	Mitochondrial	ACO2	CaOrf19.6632	MITO	ACO2	At4g26970	Mitochondrial
							ACO3	At2g05710	Mitochondrial

Proteins, corresponding genes, accession number, and targeting information are shown for enzymes carrying out activities required for the glyoxylate cycle in S. cerevisiae, C. albicans, and A. thaliana. PTS and MITO indicates predicted targeting information (general prediction: http://wolfpsort.org/, PTS1-predictor: http://mendel.imp.ac.at/pts1/, Mito-predictor: http://ihg.gsf.de/ihg/mitoprot.html). Cytosolic, peroxisomal, mitochondrial, and chloroplast indicates experimentally verified localization. Bold underlined are proteins experimentally proven to contribute to the glyoxylate cycle.

In the genome of *S. cerevisiae* the key enzymes ICL and MLS are encoded by two genes each. Based on the high expression levels when cells grow on ethanol, acetate or fatty acids and the inability of cells lacking these proteins to grow on these carbon sources Icl1p and Mls1p have been attributed to the glyoxylate cycle (Fernandez et al., 1992; Hartig et al., 1992). The two others, Icl2p and Dal7p contribute to the mitochondrial propionate metabolism and the purine degradation, respectively (Hartig et al., 1992; Luttik et al., 2000). From the three genes coding for MDH, a mitochondrial (Mdh1p; McAlister-Henn and Thompson, 1987), a cytosolic (Mdh2p; Minard and McAlister-Henn, 1991), and a peroxisomal (Mdh3p; Steffan and McAlister-Henn, 1992) gene product are derived. Only the cytosolic and the mitochondrial variants are expressed when cells grow on C_2_-carbon sources (ethanol/acetate) and the cytosolic enzyme was required for growth under these conditions (Minard and McAlister-Henn, 1991; McCammon, 1996). This experimental evidence indicates that the cytosolic Mdh2p participates in the glyoxylate cycle.

Similarly, among the three CIT proteins encoded within the yeast genome only Cit2p is considered to contribute to the glyoxylate cycle, because of its expression pattern on various carbon sources and because of the mitochondrial location of the other two enzymes Cit1p and Cit3p. Interestingly, Cit2p is essential for growth on ethanol or acetate only, when an alternative route probably involving one of the two mitochondrial activities is blocked (Van Roermund et al., 1995). Finally, one gene encoding ACO (ACO1; Gangloff et al., 1990) gives rise to the cytosolic and the mitochondrial activity (Regev-Rudzki et al., 2005) attributing Aco1p to the TCA- and the glyoxylate cycle, whereas Aco2p may be important for fermentation (Van den Berg et al., 1998).

Even more complex is the situation in *A. thaliana*. Although only one gene codes for ICL (Eastmond et al., 2000) and one for MLS (Cornah et al., 2004), eight genes encode NAD^+^ dependent MDHs, five genes code for CITs and three genes code for ACOs (summarized in Table 1). The localization, expression pattern and phenotype of the corresponding T-insertion mutants may help to discern the respective function of each individual protein. Nevertheless, the attribution of a function in the glyoxylate cycle appears difficult. Three of the five CITs, CYS1-3 harbor peroxisomal targeting information whereas CYS4 and CYS5 include mitochondrial leader peptides (Pracharoenwattana et al., 2005). Similarly, among the MDH proteins two are peroxisomal, two mitochondrial, one is observed in chloroplasts and three in the cytosol (Pracharoenwattana et al., 2007) [The Arabidopsis Information Resource (TAIR), http://www.arabidopsis.org/, Huala et al., 2001].

The expression pattern of enzymes contributing to the glyoxylate cycle is very characteristic, namely high expression after imbibition during seed germination followed by strong reduction upon postgerminative growth. Isoenzymes that are not expressed in the phase of germination can be excluded from a contribution to the glyoxylate cycle, such as CYS1 (Pracharoenwattana et al., 2005). During this developmental phase both, the β-oxidation of fatty acids and the glyoxylate cycle are equally needed and therefore the expression profiles of the corresponding genes are similar (Eastmond and Graham, 2001; Rylott et al., 2001). A detailed and precise cluster analysis of expression profiles during seedling development in soybean remained inconclusive since the expression pattern of the two key enzymes ICL and MLS did not fall into the same cluster and expression differences of the isoforms of CIT and MDH were not distinguishable (Gonzalez and Vodkin, 2007).

An unambiguous attribution of a function in the glyoxylate cycle to a distinct protein could be based on the characteristics of cells deficient in the glyoxylate cycle function. *A. thaliana* seeds lacking ICL are able to germinate, but their seedling establishment is severely impaired in the absence of light or carbohydrates offered as alternative carbon source (Eastmond et al., 2000). In other words, gluconeogenesis is compromised but lipid respiration is still active in these mutants. *A. thaliana* seeds lacking MLS, the other unique enzyme of the glyoxylate cycle, display a similar defect in seedling establishment in the absence of light or carbohydrates. Regarding the compensation of the establishment defect by light the seeds respond differently. In the absence of MLS activity a lower light dose is required to promote seedling establishment than in the absence of ICL activity (Cornah et al., 2004). The latter phenotype led to the suggestion that in the absence of MLS glyoxylate, which is produced by ICL can enter gluconeogenesis by hijacking enzyme activities from the photorespiratory pathway.

Phenotypes of plants lacking various isoforms of either CYS or MDH are less revealing to discern a specific function. During seedling establishment both peroxisomal MDHs (pMDH1 and pMDH2) and peroxisomal CITs (CYS2 and CYS3) are expressed. Seeds lacking either one of the peroxisomal activities present with disturbed fatty acid degradation (Pracharoenwattana et al., 2005, 2007). Thus, a contribution of these proteins to the glyoxylate cycle cannot be delineated from the block in seedling establishment obtained in these mutant plants, because the lack of fatty acid degradation elicits a similar block in seedling establishment. Conversely, no noticeable defect in establishment was observed for seedlings from plants lacking mitochondrial MDH excluding an essential role of these MDHs in either β-oxidation of fatty acids or the glyoxylate cycle (Tomaz et al., 2010). When the metabolic distribution of exogenously added acetate within seedlings is used as indicator for the functionality of the glyoxylate cycle, no differences between seedlings lacking the peroxisomal MDH and wild type seedlings could be observed (Pracharoenwattana et al., 2007). However, this assay shows differences in the re-routing of acetate between seedlings from wild-type plants and plants, which are blocked in the glyoxylate cycle due to lack of ICL or MLS, although the differences appear small in the absence of MLS (Cornah et al., 2004). Experiments to discern the individual roles of the three cytosolic MDH were not yet carried out. Similarly, among the three ACO proteins a function in the glyoxylate cycle has not been assigned to anyone of them (Peyret et al., 1995; Arnaud et al., 2007). Altogether, *in A. thaliana* the assignment of individual ACOs, CITs, and MDHs to the glyoxylate cycle remains open.

The situation in the yeast *C. albicans* is different. Each one of the enzymes ICL, MLS, and CIT is encoded by a single gene (Piekarska et al., 2008). In this organism the same enzyme Cit1p seems to contribute to the mitochondrial TCA-cycle and the glyoxylate cycle such as demonstrated for *Candida tropicalis* (Ueda et al., 1997). However, it cannot be excluded that a cytosolic form is derived from alternative translation as two variants are described (C4YLG7 and Q59ZZ5), but none of the described variants contains a peroxisomal targeting signal type 1 (PTS1; C-terminus KYIELVKNINKA). ACO activity is encoded by two genes, MDH activity by three genes (Jones et al., 2004). In each case one of the variants has a closer similarity to the respective glyoxylate cycle enzyme in *S. cerevisiae*, but experimental evidence for their role is missing.

### Subcellular localization of the enzymes of the glyoxylate cycle

In *S. cerevisiae* the enzymes contributing to the glyoxylate cycle are distributed between the peroxisomal matrix and the cytosol. Icl1p, Mdh2p, and Aco1p were described in the cytosol (Minard and McAlister-Henn, 1991; Taylor et al., 1996; Regev-Rudzki et al., 2005), and Cit2p was found in the peroxisomal fraction (Lewin et al., 1990). Mls1p is either targeted to peroxisomes when cells utilize oleic acid or distributed across the cytosol when cells utilize ethanol (McCammon et al., 1990; Kunze et al., 2002). Upon growth on ethanol or acetate as sole carbon source with all other glyoxylate cycle enzymes in the cytosol the extra-peroxisomal fraction of Cit2p might suffice to generate four-carbon units through the glyoxylate cycle.

In *A. thaliana* the key enzymes ICL and MLS are considered peroxisomal containing a C-terminal PTS1 [EGTSLVVAKSRM for ICL and IVAHYPINVSRL for MLS, Arabidopsis subcellular database (SUBA), (Heazlewood et al., 2007)]. In contrast, ACOs lack peroxisomal targeting information and were allocated to the cytosol and the mitochondria (Arnaud et al., 2007). The extra-peroxisomal location of ACO activity was confirmed in castor bean and potato (Courtois-Verniquet and Douce, 1993). Which of the other gene products, namely of MDH and of CIT, are contributing to the glyoxylate cycle is unclear. Among the MDHs it can be assumed that one or more of the three cytosolic isoforms participates in the glyoxylate cycle as mutant plants lacking either the peroxisomal (Pracharoenwattana et al., 2007) or the mitochondrial (Tomaz et al., 2010) isoenzymes show no signs of a glyoxylate cycle defect.

In *C. albicans* both key enzymes of the glyoxylate cycle, ICL and MLS were found in peroxisomes irrespective of the carbon source used (Piekarska et al., 2008), rendering this microorganism an alternative yeast model with key enzyme distribution similar to *A. thaliana*. However, the exclusive presence of one citrate synthase gene (CIT1) and the necessity of a peroxisomal and mitochondrial shuttle mechanism for acetyl-CoA for growth on oleic acid indicates that peroxisomal CIT is not available, but a cytosolic isoform might well be. The similarity of the MDH proteins to the homologs from *S. cerevisiae* suggests that the cytosolic Mdh2p contributes to the glyoxylate cycle as well. Finally, the cytosolic ACO Aco1p (Jones et al., 2004) might be supplemented by a minor peroxisomal subfraction that is caused by a weak PTS1 [HGSALNFIKSKY, http://mendel.imp.ac.at/pts1/, Neuberger et al., 2003].

Briefly summarized, the localization of proteins participating in the glyoxylate cycle on different sides of the peroxisomal membrane in all three model organisms requires the transport of intermediates across the lipid barrier.

## Transfer of metabolites

Usually, the concentration of free intermediates occurring in metabolic pathways is relatively low, because a local accumulation of participating enzymes, e.g., within an organelle, or a physical interaction between successive enzymes allows a channeling of intermediates. In extreme cases, large protein complexes such as the fatty acid synthase transfer small molecules from one active center to the next. If a membrane separates consecutive steps an efficient metabolic pathway requires a direct shuttling of intermediates either through a transporter protein or through a proteinaceous channel linking enzymes on both sides of the membrane. Alternatively, a comparably high net concentration of intermediates might facilitate diffusion controlled transfer, which appears unlikely for highly reactive compounds such as glyoxylate.

### Permeability of the peroxisomal membrane

To seal certain reactions in a compartment and to restrict the generation of reactive molecules cells seem to spare no effort to translocate the corresponding proteins and metabolic precursors into distinct compartments. The localization of various oxidases to peroxisomes is a typical example for an energy spending activity preventing contamination of the cytosol by H_2_O_2_. In turn, the membrane delimiting a compartment thought to protect the cellular interior from detrimental effects by small, highly reactive molecules could be expected to be impermeable for such substances. However, early studies provided evidence for a permeability of the peroxisomal membrane for small solutes such as urate or amino acids and for density gradient material such as sucrose (De Duve and Baudhuin, 1966; Van Veldhoven et al., 1987). For cofactors and larger substrates required for peroxisomal enzymes such as NAD or acetyl-CoA the peroxisomal membrane was shown to act as barrier similar to the inner mitochondrial membrane (Van Roermund et al., 1995). Shuttle systems were proposed to functionally connect the peroxisomal lumen with the cytosol exchanging substrates and keeping the cofactors in the reduced or in the oxidized state as required (Elgersma et al., 1995; Van Roermund et al., 1995; Antonenkov and Hiltunen, 2006; Visser et al., 2007).

A concept for the peroxisomal membrane permeability reconciling conflicting data was put forward by Hiltunen and co-workers (Antonenkov et al., 2004a,b). Pore-like structures permit the free exchange of small solutes (MW < 300D) across an otherwise impermeable membrane inhibiting the transfer of molecules such as acetyl-CoA, ATP, or NAD. Accordingly, pore-forming activities were reported in peroxisomes isolated from plants, mammalian tissue and yeast (Reumann et al., 1995; Antonenkov et al., 2005; Grunau et al., 2009), but the molecular nature of the channel proteins remained largely unknown. In contrast, specific transporters for adenine nucleotides and NAD were identified corroborating the impermeability of the peroxisomal membrane for such bulky molecules (Palmieri et al., 2001; Bernhardt et al., 2012; for reviews see Antonenkov and Hiltunen, 2012; Hu et al., 2012).

The localization of enzymatic activities of the glyoxylate cycle on different sides of the peroxisomal membrane requires an efficient transport of intermediates across the lipid barrier (Kunze et al., 2006). When acetyl-CoA is generated inside the peroxisomal matrix—usually via β-oxidation of fatty acids—it remains confined to peroxisomes probably because of its size (Van Roermund et al., 1995; Antonenkov and Hiltunen, 2006). CIT catalyzes the condensation of acetyl-CoA with oxaloacetate into citrate, which in turn is exported and serves as substrate for extra-peroxisomal ACO. The resulting isocitrate is imported into peroxisomes in those organisms in which the corresponding cleavage activity, ICL resides inside peroxisomes (*A. thaliana, C. albicans*). Thereby, succinate, the net product of the cycle is released within peroxisomes and requires an additional export mechanism. Conversely, glyoxylate is directly handed over to the second acetyl-CoA consuming enzyme, MLS, which is a peroxisomal constituent in all organisms.

Some organisms such as *S. cerevisiae* do not harbor a peroxisomal ICL (Taylor et al., 1996), but instead generate the products of this reaction in the cytosol. The disadvantage that a small reactive molecule such as glyoxylate needs to be translocated across a membrane to reach MLS might be balanced by the advantage that succinate is already in the cytosol, which is one step closer to its usual final destination, mitochondria. The interesting observation that the relevant MDH activity for the glyoxylate cycle is exerted by cytosolic isoforms in the yeast *S. cerevisiae* and supposedly also in *A. thaliana* calls for an additional export of malate and the subsequent import of oxaloacetate to close the cycle (Minard and McAlister-Henn, 1991; Pracharoenwattana et al., 2007).

**Figure 2:**
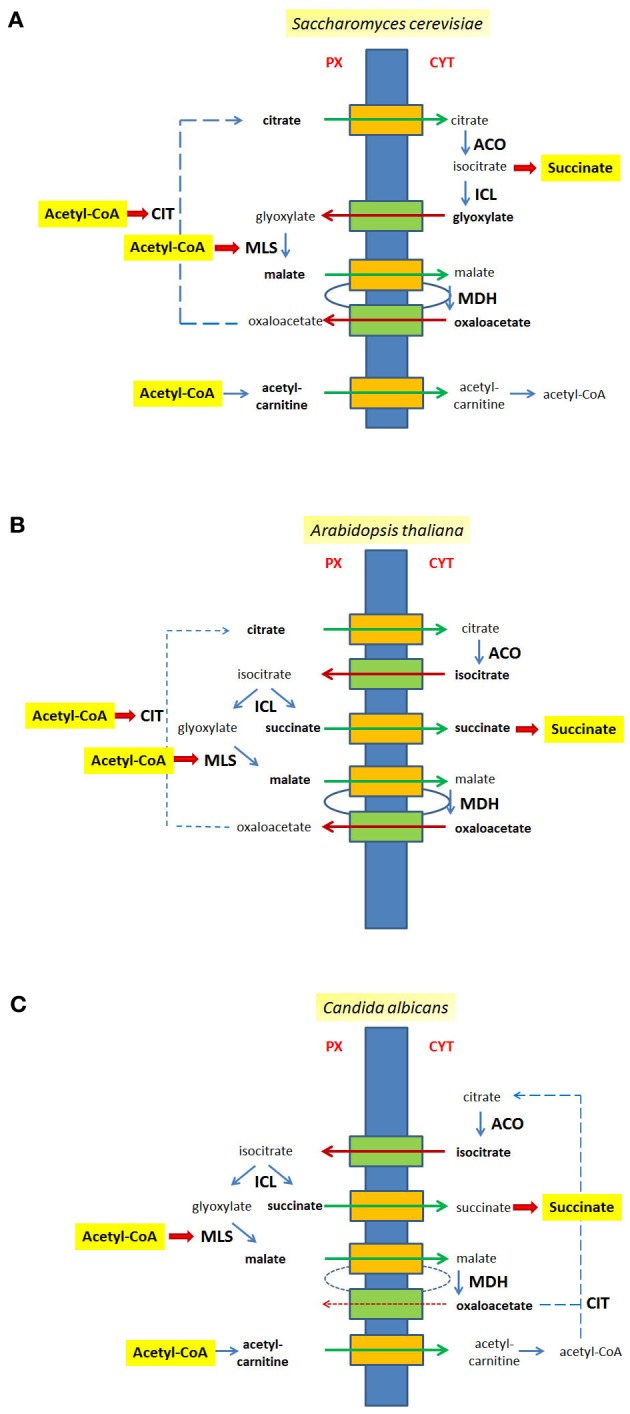
**Metabolites of the glyoxylate cycle crossing the peroxisomal membrane.** The peroxisomal membrane facilitates the transfer of small metabolites. The transport of glyoxylate cycle intermediates and of C_2_-units is shown for *S. cerevisiae* **(A)**, *A. thaliana* **(B)**, and *C. albicans* **(C)**. Hypothetical pore-forming proteins permitting the export of intermediates are colored orange, hypothetical pore-forming proteins permitting the import of intermediates are colored green. Broken lines are drawn to close the glyoxyate cycle. Intermediates that cross the peroxisomal membrane are indicated bold. *Px*, peroxisomal side of the membrane, *Cyt*, cytosolic side of the membrane.

All in all a series of transport steps is required to complete a full round of the glyoxylate cycle. If ICL is extra-peroxisomal, e.g., in the yeast *S. cerevisiae*, citrate and malate are exported, and glyoxylate and oxaloacetate are imported (Figure 2A). If ICL resides inside peroxisomes citrate, malate and succinate are exported, whereas isocitrate and oxaloacetate are imported (Figure 2B), a situation occurring in the plant *A. thaliana*. A similar flux of intermediates occurs in *C. albicans*, although citrate is generated in the cytosol and does not occur in peroxisomes (Figure 2C).

The export of malate and import of oxaloacetate as part of the glyoxylate cycle mimics a redox-shuttle system consisting of a peroxisomal and a cytosolic MDH activity that mediates a net-export of reduction equivalents generated by the β-oxidation of fatty acids to the cytosol. Thus, peroxisomal malate generated by MLS might be fueled into the same path to the cytosol as malate generated by the re-oxidation of NADH (peroxisomal *Sc*Mdh3p or *At*pMDH1/pMDH2). Similarly, the transport of isocitrate might be part of a shuttle mechanism coupling the import of isocitrate and the export of α-oxo-glutarate with the reduction of NADP^+^ inside peroxisomes. Such a hypothetical net-exchange is based on a cytosolic isocitrate dehydrogenase (*Sc*Idp2p) and a peroxisomal isoform (*Sc*Idp3p) converting isocitrate into α-oxo-glutarate and CO_2_ and *vice versa*.

The export of citrate is not restricted to the glyoxylate cycle but also presents a mode of export for C_2_-units that are generated as acetyl-CoA by fatty acid β-oxidation within peroxisomes. In this case the export of citrate is ultimately balanced by the import of oxaloacetate, the precursor for CIT. The export of citrate is considered the only export pathway for C_2_-units in plants since lack of the peroxisomal CITs, CYS2 and CYS3, blocks β-oxidation of fatty acids in *A. thaliana* (Pracharoenwattana et al., 2005), whereas in the yeast *S. cerevisiae* an additional export system for acetyl-units exists. For the latter, a peroxisomal form of carnitine acetyl-transferase (Cat2p; Elgersma et al., 1995) generates acetyl-carnitine that can be translocated across the membrane and rebuilt into acetyl-CoA either in the cytosol or within mitochondria employing either one of three carnitine acetyltransferases, Cat2p, Yat1p, or Yat2p (Swiegers et al., 2001). In *S. cerevisiae* the β-oxidation of fatty acids requires only one mode of export and thus the lack of either the peroxisomal CIT Cit2p or the carnitine acetyltransferase Cat2p localized to the peroxisomes and the mitochondria is tolerated for growth on oleic acid as carbon source, but upon deletion of both genes (ΔCIT2ΔCAT2) cells were not able to utilize oleate (Van Roermund et al., 1999). Moreover, under these conditions the cytosolic re-conversion of acetyl-carnitine into acetyl-CoA is also essential, as mutant cells lacking CIT2 and either one of the other carnitine acetyl-transferases (YAT1 or YAT2) were unable to consume oleic acid (Swiegers et al., 2001). In *C. albicans* a peroxisomal condensation of acetyl-CoA with oxaloacetate producing citrate is not possible due to the lack of a peroxisomal CIT. Therefore, the export of acetyl-units depends on the carnitine form and consequently, in the absence of the peroxisomal isoform of Cat2p cells were unable to grow on oleic acid (Strijbis et al., 2010).

The transfer of the various small intermediates is compatible either with specific transporter proteins or with atypical permeability properties of the membrane. The mitochondrial and chloroplast membranes contain numerous transporter proteins specific for small organic compounds, but corresponding proteins were not identified in peroxisomes. Few examples may illustrate this. In mitochondria of *S. cerevisiae* a carnitine acylcarnitine carrier protein Crc1p and a citrate-oxoglutarate carrier were identified, but no peroxisomal paralog was discovered yet (Van Roermund et al., 1999; Castegna et al., 2010). Similarly, a succinate-fumarate transporter was identified in the inner mitochondrial membranes of *S. cerevisiae* and *A. thaliana* [Sfc1p/Acr1p (Bojunga et al., 1998) and AtMSFC1 (Catoni et al., 2003), respectively], but no homologous protein was identified to mediate plant peroxisomal succinate export. Furthermore, a glycolate/glycerate transporter required for photorespiration (PLGG1) has been identified in the chloroplast membrane (Pick et al., 2013), but no homologous proteins were found in plant peroxisomes.

Despite overwhelming evidence for the free exchange of small solutes the molecular nature of pore-forming activities in plant and yeast peroxisomes remained unknown (Reumann et al., 1997, 1998; Grunau et al., 2009). Moreover, the yeast peroxisomal membrane pores were shown to conduct solutes of the glyoxylate cycle (Antonenkov et al., 2009; Grunau et al., 2009). So far, only in mammalian peroxisomes a protein, Pxmp2, was identified that exhibits channel-forming capacities (Rokka et al., 2009).

Provided that the transport of intermediates is essential for the metabolic activity, the lack of such pores should result in non-functional peroxisomes. Therefore, the corresponding genes should have turned up in various genetic screens searching for mutants with dysfunctional peroxisomes. However, more than one gene could encode redundant functions escaping the detection of these genes in screens. Likewise, such porins should be rather abundant constituents of the peroxisomal membrane and as such should have been identified in various proteomic approaches. The most abundant yeast peroxisomal membrane protein reported is Pex11p, but its localization at the outer surface renders a function in solute transport rather unlikely (Erdmann and Blobel, 1995; Van Roermund et al., 2000; Opalinski et al., 2011).

## Bipartite enzyme distribution of glyoxylate cycle enzymes: subcellular distribution follows generation of substrates

The hypothesis that substrate availability was the driving force for changes in enzyme localization, has been proposed based on observations in the yeast *S. cerevisiae*. In this organism Mls1p localization differs between cells grown in medium containing ethanol as sole carbon source, when acetyl-CoA is generated primarily in the cytosol, and cells utilizing oleic acid generating acetyl-CoA within peroxisomes (Kunze et al., 2002, 2006).

In other organisms such as the plant *A. thaliana* ICL and MLS are peroxisomal. In this case an efficient coupling of the glyoxylate cycle to β-oxidation of fatty acids is essential, because both, energy and biomass production for germination and seedling outgrowth rests on the β-oxidation of fatty acid. The resulting acetyl-CoA is used to generate energy via the TCA-cycle and oxidative phosphorylation and to feed the glyoxylate cycle to cover the needs for biosynthetic processes. For the opportunistic fungus *C. albicans* carbohydrates and fatty acids are the prevalent carbon sources in its natural habitat, the mammalian gut. Thus, acetyl-CoA is expected to be obtained primarily inside mitochondria following glycolysis or inside peroxisomes via β-oxidation under such conditions. Even when these cells were grown in the presence of ethanol or acetate both key enzymes (ICL and MLS) are located in peroxisomes (Piekarska et al., 2008). Needless to mention, that neither *C. albicans* nor plants ever face ethanol or acetate as carbon source in their natural environment. This supports the hypothesis that the prevailing source of acetyl-CoA under natural conditions determines the localization of glyoxylate cycle key enzymes. For both model systems neither evolutionary pressure nor man-made selection forced the organism to change the intracellular location of parts of the glyoxylate cycle.

In contrast, strains of baker's yeast, *S. cerevisiae*, have been selected for efficient growth in the presence of ethanol. Consequently, this organism most efficiently utilizes acetyl-CoA generated from this carbon source in the cytosol, which is apparently supported by the potential to relocate the complete glyoxylate cycle to the cytosol.

The bipartite localization of enzymes provokes unusual intricacies for a straightforward metabolic pathway consisting of five enzymatic activities. Evolutionary optimization and pressure to increase efficiency demand a physiological advantage to compensate for this complexity. We suggest that the distribution of the glyoxylate cycle enzymes on different sides of the peroxisomal membrane might be due to the combination of (i) the unavoidable provision of its substrate, acetyl-CoA, inside peroxisomes by fatty acid β-oxidation (Kunze et al., 2006) and (ii) the incompatibility of some of its enzymes, namely ACO (Verniquet et al., 1991) and to a certain extent also ICL (Yanik and Donaldson, 2005), with the oxidative milieu within peroxisomes including high H_2_O_2_ concentrations. Thus, during evolution neither compartmentation of the complete pathway within peroxisomes nor the transfer of all enzymes into the cytosol appeared as feasible alternative.

However, the tight coupling of peroxisomal acetyl-CoA generation and its fueling into the glyxoylate cycle that appears optimal for growth of *A. thaliana* and *C. albicans* in their natural habitats might restrain the incorporation of extra-peroxisomal acetyl-CoA into the glyoxylate cycle. When such organisms utilize the less physiological carbon source acetate as carbon source they satisfy all their energetic and biosynthetic needs from acetyl-CoA that is primarily generated in the cytosol. In this case the peroxisomal membrane could act as barrier that separates cytosolic acetyl-CoA from those peroxisomal enzymes of the glyoxylate cycle that utilize it, namely MLS in both organisms and CIT in *A. thaliana*. Under these conditions the mitochondrial energy production from cytosolic acetyl-CoA is not expected to be limited, but the biosynthetic capacity of the glyoxylate cycle is restricted by the transfer of acetyl-CoA into peroxisomes involving a specific import mechanism for this intermediate. Such a limitation is avoided in *S. cerevisiae*, where the glyoxylate cycle can be relocated to the cytosol.

Interestingly, *A. thaliana* and *C. albicans* can grow under conditions when acetate is utilized as sole carbon source (Hooks et al., 2004; Zhou and Lorenz, 2008), but they are dependent on specific peroxisomal functions. In *A. thaliana* a genetic screen for mutants that are unable to utilize exogenous acetate identified two genes encoding peroxisomal proteins (Hooks et al., 2004). The peroxisomal transporter protein *COMATOSE* known to transport fatty acids (Hooks et al., 2007) and the intraperoxisomal short chain acyl-CoA/acetyl-CoA synthase (At3g16910, AAE7) (Turner et al., 2005; Shockey and Browse, 2011) are required for the integration of acetate into organic compounds (Allen et al., 2011). Interestingly, neither *C. albicans* nor *S. cerevisiae* cells require functional peroxisomes for the utilization of acetate, as cells harboring a deletion of a PEX-gene can grow on medium solely containing this carbon source (Piekarska et al., 2008). However, upon selective interruption of fatty acid β-oxidation by the ablation of the enzyme exerting the second step (Fox2p), *C. albicans* cells cannot utilize acetate any more, but *S. cerevisae* cells can do so (Hiltunen et al., 1992; Piekarska et al., 2008). This phentotype of *C. albicans* cells might be due to their inability to feed cytosolic acetyl-CoA into the glyoxylate cycle, because MLS is enclosed by the peroxisomal membrane. This notion is supported by the observation, that the ability to utilize acetate efficiently can be restored in these cells (ΔFOX2), when the compartmentation of peroxisomal enzymes is prevented by the deletion of the PTS1-receptor PEX5 (ΔFOX2ΔPEX5) (Piekarska et al., 2008). Thus, in organisms optimized for an efficient consumption of fatty acids the redirection of cytosolic acetyl-CoA units obtained from other sources such as acetate into peroxisomes is a critical step for its fueling into the glyoxylate cycle.

## Metabolic fluxes in related metabolic pathways

**Figure 3:**
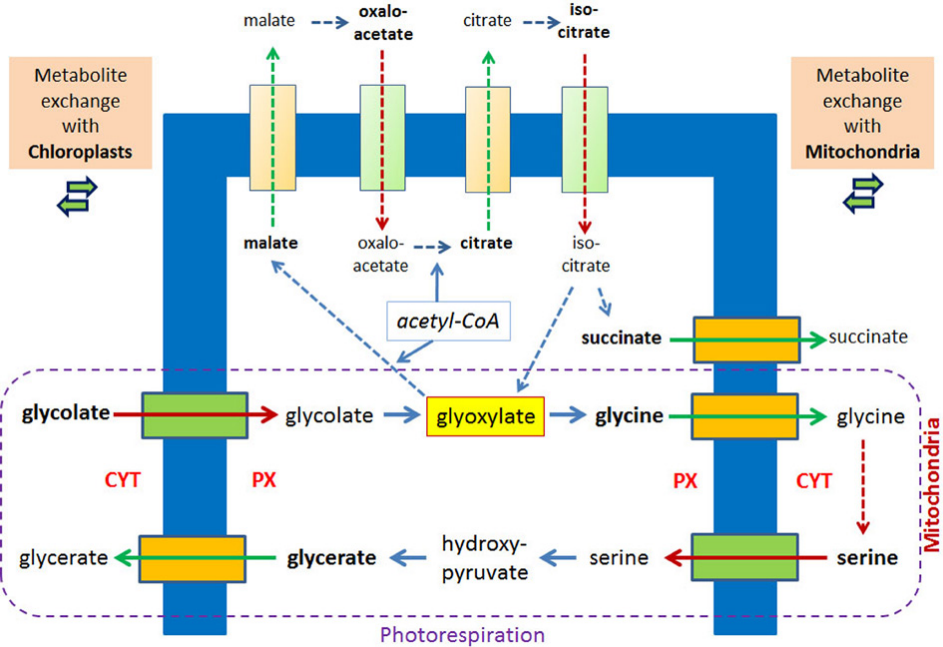
**Metabolite transport processes for the glyoxylate cycle and the photorespiratory process are considered to be similar.** In plant peroxisomes glyoxylate is a key metabolite of the glyoxylate cycle and the photorespiration, the latter involving mitochondria and chloroplasts. Hypothetical pore-forming proteins permitting the export are colored orange, hypothetical pore-forming proteins permitting the import are colored green. The broken lines with arrow heads indicate reactions of the glyoxylate cycle and the conversion of two molecules of glycine into serine inside mitochondria without further details. Reactions of the photorespiration process are encircled.

Photorespiration represents another example for a metabolic process in which glyoxylate is formed and intermediates traverse the peroxisomal membrane. Considering only the transport reactions between chloroplasts and peroxisomes two molecules of glycolate are transported into peroxisomes and one molecule glycerate is exported. The exchange with the mitochondria includes the export of two molecules of glycines and the import of one molecule serine (Figure 3). Already 40 years ago peroxisomes, mitochondria, and chloroplasts were often seen in close vicinity in the electron microscope (Frederick and Newcomb, 1969). A direct apposition of the peroxisomal membrane and the outer membranes of chloroplasts (Schumann et al., 2007) supports the hypothesis of direct exchange of metabolites thereby avoiding diffusion of intermediates into the cytosol. In leaves of *A. thaliana*, in which due to a mutation in a peroxisomal membrane protein the close apposition of chloroplasts and peroxisomes is lost, glyoxylate accumulates (Schumann et al., 2007).

Under normal conditions the glyoxylate cycle and the photorespiration do not overlap although they occur in the same organelle and share glyoxylate as key intermediate. This separation is due to the developmental program, which shows highest glyoxylate key enzyme activities during seed germination [2–4 days postimbibition (Eastmond and Graham, 2001)], whereas the enzymes of the photorespiration are induced by light (Bertoni and Becker, 1993) upon cotyledon development. Upon this development the change of glyoxylate cycle containing peroxisomes (“glyoxysomes,” see Pracharoenwattana and Smith, 2008) into photorespiratory active peroxisomes is considered prototypical for the exchange of enzymatic equipment of such organelles (Titus and Becker, 1985; Behrends et al., 1990) and is even accompanied by the highly surprising removal of ICL from “glyoxsomes” (Lingard et al., 2009). However, employing artificial experimental conditions by exposing outgrowing seedlings to light these developmental programs become blended, and so does the enzymatic equipment. This is demonstrated by the comparison of plants in which the glyoxylate cycle is interrupted either by ICL deficiency or MLS deficiency. The observed growth defect of these plants in the dark can be completely overcome by exposing seedlings lacking MLS to light, but seedlings lacking ICL can only partially recover (Cornah et al., 2004). The suggestion that the addition of photorespiratory equipment allows the re-direction of glyoxylate from MLS to serine/glutamine-glyoxylate aminotransferase is supported by the re-organization of the incorporation pattern of radioactively labeled acetyl-CoA into different water soluble intermediates. Thereby, wild type plants and plants lacking MLS display a more similar phenotype than plants lacking ICL.

## Model for the flux of intermediates across a membrane

The localization of enzymes catalyzing successive metabolic reactions on opposite sides of the peroxisomal membrane and the permeability of this membrane for small molecules raises the question how fast small metabolites can cross the barrier membrane. Such a transfer of intermediates might be accomplished either (i) by an unspecific pore or (ii) by a series of specific transporter or facilitator proteins or (iii) by the direct coupling of specific enzymes on both sides of an unspecific pore forming a transmembrane metabolon. A simple diffusion controlled mechanism involving only a pore-like structure appears unlikely, because after reaching the other side of the membrane small metabolites could easily diffuse and the concentration might become (quite) low. This may happen, permitting the adjustment of concentrations between the cytosol and the peroxisomal matrix. However, such a model appears less likely for instable, highly reactive or even toxic intermediates such as glyoxylate. Alternatively, specific transporters or facilitators might close the gap between successive enzymatic reactions and guarantee specificity of transport, however these proteins have not been found so far. An interesting alternative might be a transmembrane metabolon comprised of transiently accumulating metabolic enzymes such as the glyoxylate cycle enzymes on both sides of an unspecific pore. We propose that in this extended form of the classical metabolon (Srere, 1987) pore-like channels participate in the assembly of supramolecular complexes, thereby linking proteins on both sides of the membrane enabling the swift transfer or exchange of metabolites. Such structural arrangement could enhance efficiency and ensure sufficient flux of intermediates across the peroxisomal membrane. Importantly, the transiently formed transmembrane metabolon would generate only local concentration peaks of intermediates at the entry sides of the pores. In our model metabolically active proteins transiently acquire a pore leading to a rapid transfer of the corresponding metabolites across a short distance. In this respect the observation seems interesting that polyethylene glycol (PEG), which stabilizes peroxisomal membrane integrity during the isolation procedure (Antonenkov et al., 2004a) is also known as stabilizing agent for supramolecular protein complexes such as metabolons (Beeckmans and Kanarek, 1981; Barnes and Weitzman, 1986). Such a model would explain how a variety of substrates could cross the membrane without specific transporter molecules. The selectivity would be maintained via the transient association of proteins on both sides of the pore-like channels providing control and efficiency.

According to our model in *S. cerevisiae* the loosely associated protein complex outside peroxisomes would consist of ACO, ICL, and MDH, on the inside of MLS and CIT, and the channel(s) would allow transferring citrate and malate from the matrix to the cytosol and/or glyoxylate and oxaloacetate from the cytosol to the peroxisomal matrix. When the peroxisomal Mdh3p has no access to the complex formed it is excluded from participation in the glyoxylate cycle. In plants, five metabolites have to cross the membrane. ACO and MDH acting outside peroxisomes require the export of citrate and malate and the import of isocitrate and oxaloacetate for further processing. The product succinate needs to be exported as well to make it available for the cellular metabolism. Since according to our model ICL would loosely associate with CIT and MLS it is reasonable to suggest that the simultaneous export of succinate using the same channel unit represents an additional drive for the cleavage reaction.

Furthermore, the peroxisomal membrane would *per se* not represent a principal barrier for glyoxylate cycle intermediates or any other small metabolite, but controls the velocity of the continuous flux. In addition, this model could also explain how shuttles may function. A local concentration of enzymes on both sides of a membrane-spanning pore could facilitate the shuttling of reduction equivalents in [isocitrate/α-oxo-glutarate (Van Roermund et al., 1998)] or out [malate/oxaloacetate (Van Roermund et al., 1995), lactate/pyruvate (Baumgart et al., 1996), G3P/DHAP (Gee et al., 1974)] of the peroxisomal matrix (for review see Antonenkov and Hiltunen, 2006). The exchange of the reduced and oxidized intermediates would occur due to a local association of the corresponding enzymes, Mdh2p/Mdh3p or Idp2p/Idp3p, on each side of a channel.

## Conclusions and perspectives

The peroxisomal matrix is surrounded by a single membrane that allows the enclosure of a variety of highly reactive and even toxic compounds. However, *in vitro* the membrane appears permeable to small molecules and neither a chemical nor an electrochemical gradient has been detected, which represents a major difference to the mitochondrial inner membrane or the thylakoid membrane. The biophysical properties of the peroxisomal membrane demands pore-like structures in addition to the few transmembrane proteins with transporter function for larger molecules. The frequent exchange of intermediates between peroxisomes and the intracellular environment requires directed transport processes with high selectivity, which appears incompatible with pore-like exchange modules. However, a model in which soluble enzymes accumulate in proximity to both ends of such pore-like structures forming a transmembrane metabolon would explain rapid and selective exchange based on a local increase in intermediate concentration. The observation that the enzymes of the glyoxylate cycle are distributed across both sides of the peroxisomal membrane would be prototypical for such transport processes, because an efficient transfer of small organic metabolites across the peroxisomal membrane is essential for this pathway. The high variability in the nature of small organic molecules transported across the peroxisomal membranes between *S. cerevisiae*, *C. albicans*, and *A. thaliana* may reflect the ease of adaptation processes whenever specific transporter proteins are not involved. Similar considerations are applicable to the photorespiration, although the direct exchange of metabolites with other organelles renders the latter apparently more complex. A transport system of such high versatility generates an organelle of high plasticity and allows rapid adjustments to environmental changes and to developmental programs generally considered an important feature of peroxisomes.

Altogether, the peroxisomal membrane markedly differs from membranes of other metabolically active organelles, which might represent an intermediate step in the development of an organelle with novel properties. Thus, it can serve as model system to investigate and to understand transport processes across membranes with reduced complexity.

### Conflict of interest statement

The authors declare that the research was conducted in the absence of any commercial or financial relationships that could be construed as a potential conflict of interest.
